# Dissolving microneedle patch-assisted transdermal delivery of methotrexate improve the therapeutic efficacy of rheumatoid arthritis

**DOI:** 10.1080/10717544.2022.2157518

**Published:** 2022-12-19

**Authors:** Weiman Zhao, Lijie Zheng, Jianhui Yang, Zihui Ma, Xinyi Tao, Qingqing Wang

**Affiliations:** a School of Pharmacy, Bengbu Medical College, Bengbu, China; b School of Pharmacy, Anhui Medical University, Hefei, China; c Engineering Research Center for Biochemical Pharmaceuticals of Anhui Province, Bengbu Medical College, Bengbu, China

**Keywords:** Dissolving microneedle patch, methotrexate, rheumatoid arthritis, transdermal delivery

## Abstract

Methotrexate (MTX) is a first-line treatment for rheumatoid arthritis (RA), but its clinical use is greatly limited by the adverse effects and poor patient compliance caused by traditional oral administration or injection. In recent years, some transdermal drug delivery systems have received considerable attention due to overcoming these shortcomings. In this study, we developed dissolving microneedle patch (DMNP) for transdermal delivery of MTX to treat RA safely and effectively. The morphology, mechanical strength, skin insertion, drug content, in vitro transdermal delivery, and other properties of DMNP were characterized. Meanwhile, the adjuvant-induced arthritis model of rats was established to investigate the therapeutic effect of MTX-loaded DMNP in vivo. The results showed that the microneedles had excellent morphology with neat array and complete needles, good puncture performance and mechanical strength, and rapid intradermal dissolution rate. In vitro transdermal delivery results indicated that microneedles could significantly increase drug transdermal permeation compared with the cream group. The pharmacological study showed that MTX-loaded DMNP significantly alleviated paw swelling, inhibit inflammatory response via downregulating the levels of TNF-α and IL-1β, relieved synovium destruction with less cartilage erosion, and slowed the progression of RA in AIA rats. Besides, DMNP presented better therapeutic performance than cream or intragastric administration at the same dosage of MTX. In conclusion, the MTX-loaded dissolving microneedle patch has advantages of safety, convenience, and high efficacy over conventional administrations, laying a foundation for the transdermal drug delivery system treatment of rheumatoid arthritis.

## 1. Introduction

Rheumatoid arthritis (RA), a chronic and systemic autoimmune disease, is always accompanied by pathological features of synovium hyperplasia, articular cartilage defects, and inflammatory cell infiltration (Guo et al., 2018), seriously affecting the lives of patients. The drugs for the treatment of RA are classified as nonsteroidal anti-inflammatory drugs (NSAIDs), glucocorticoids, and disease-modifying antirheumatic drugs (DMARDs) (Gorantla et al., 2020). With the common feature of modifying and slowing the progression, DMARDs can suppress joint swelling and pain, reduce acute markers, and improve function.

Methotrexate (MTX), representative of chemical DMARDs, has been the anchor drug and the gold standard in RA (Wilsdon & Hill, 2017). It can effectively relieve arthritis symptoms and control the progress of disease either used alone (Lucas et al., 2019) or combination with other chemical or biological DMARDs (Dale et al., 2007; Becciolini et al., 2016). Although the exact anti-rheumatoid mechanism of MTX has not been completely clarified, it is considered the action is involved in inhibiting proinflammatory cytokines generation (like IL-1β and TNF-α) (Gerards et al., 2003; Shiozawa et al., 2016) and purines or pyrimidines synthesis (Mitragotri & Yoo, 2011), promoting the shift of T helper (Th1) cells-to-Th2 and suppressing transmethylation reactions (Song et al., 2010).

Despite MTX significantly improving the condition of RA, the present administration routes are not satisfactory. The most commonly used drug delivery system clinically is oral administration, but it brings severe first pass elimination and poor bioavailability. The long-term use of MTX orally may lead to minor adverse effects (e.g. nausea and gastrointestinal bleeding) and serious effects (e.g, bone marrow depression and hepatotoxicity) (Kalb et al., 2009; Khan et al., 2012; Wang et al., 2018), which often results in more than half of patients discontinuing or switching to expensive biological agents (Bello et al., 2017; Rohr et al., 2017). Though the injection of MTX can increase the bioavailability and avoid the systemic adverse effects, the long-term and frequent injections may also reduce patients compliance and even induce infections (Bechard et al., 2014; Jacobse et al., 2019). Transdermal drug delivery system (TDDS) has recently attracted attention for the advantages, such as evading systemic toxicity especially gastrointestinal reaction and avoiding the pain or infection of injection. Nonetheless, the transdermal passive diffusion of MTX is limited because of its hydrophilicity, the barrier of *stratum corneum* (SC), and the ion form at physiological pH (Trotta et al., 2004; Dos Santos et al., 2017). To address the problem, some technologies have been studied including penetration enhancers such as liposomes, nanogels (Singka et al., 2010; Feng et al., 2018), electroporation (Wong et al., 2005), and ultrasound (Nguyen & Banga, 2018b) to promote the percutaneous delivery of MTX. In spite of the permeability increase, there are still challenges as it may damage the skin, need additional equipment or professional operators, and the precise dose of MTX is difficult to administrate. Therefore, it is highly essential to establish a safe and effective drug delivery system of MTX for RA treatment.

Microneedles (MNs), composed of single or multiple needles at the height of 25–2000 μm attached to a supporting base patch (Donnelly et al., 2010), have attracted wide attention. MNs can not only penetrate the SC, create drug delivery channels, and increase the permeability of drugs effectively, but also avoid touching the nerves and blood vessels in the dermis by adjusting the size of needles, so as to reduce or eliminate pain and infection. Some studies have adopted solid microneedles only to form drug delivery channels, and then they were removed to apply MTX (Abla et al., 2013; Nguyen & Banga, 2018a). Nevertheless, the studies were limited to in vitro research and had complicated preparation process. Besides, the two-step strategy is considered cumbersome and error-prone (Ita, 2015), and the channels will close quickly. Dissloving microneedles (DMNs), consisting of biodegradable water-soluble polymer, can overcome the drawbacks. Unlike other kinds of microneedles (coated, metal or hollow MNs), DMNs are safer for dissolving rapidly in the skin and releasing the encapsulated drugs without production of biohazardous waste and have larger loading capacity (Zhang et al., 2021).

**Figure 1.**
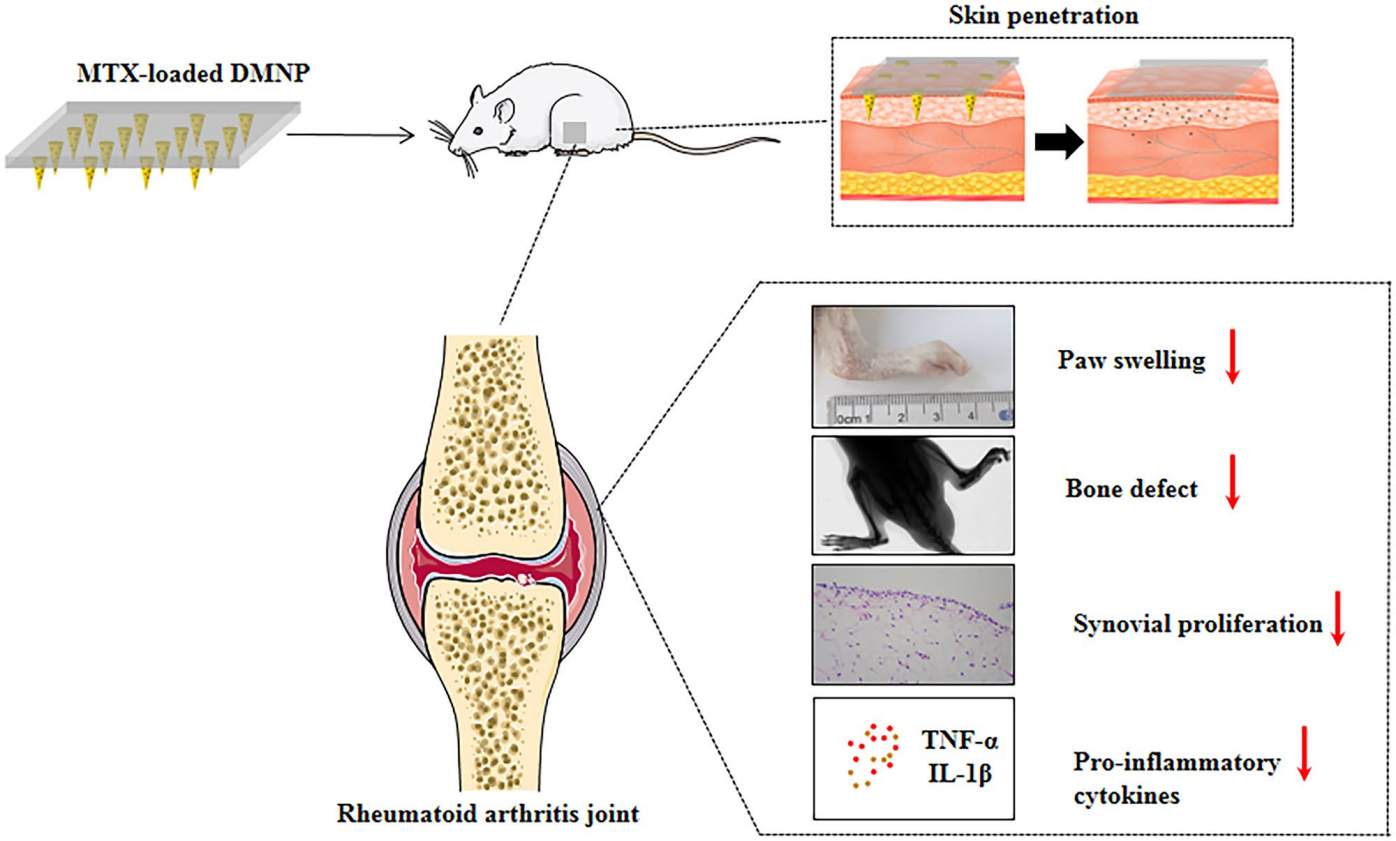
Graphical abstract of the present study. AIA rats were treated with the MTX-loaded DMNP to inhibit RA progression.

In this article, we developed a dissolving microneedle patch with the needle material of hyaluronic acid (HA) and matrix material of polyvinyl pyrrolidone (PVP K90), encapsulating MTX for the treatment of RA efficiently and safely. The DMNP was systematically characterized, including drug content, skin insertion, and in vivo dissolution. In addition, the therapeutic efficacy of DMNP on rheumatoid arthritis was observed in adjuvant-induced arthritis (AIA) rats through evaluating paw swelling, radiological change of ankle joint, histological analyses, and cytokines level in serum. The MTX–HA dissloving microneedle patch introduced in this article, a safe, effective, and convenient administration strategy of MTX, has great clinical application prospect in the treatment of RA (Figure 1).

## 2. Materials and methods

### 2.1. Materials

PVP K90 was purchased from BASF (Germany). Methotrexate (MTX, Mw = 454.44 Da) (purity >98%) was obtained from Heowns OPDE Biochemical Technology Co. Ltd. (Tianjin, China). HA was purchased from Huaxi Biotechnology Co. Ltd. (Jinan, China). Dextran (Dex) was provided by Thermo Fisher Scientific (USA). Bacillus Calmette-Guerin was purchased from the Chengdu Institute of Biological Products Co., Ltd (Sichuan,China). Trypan Blue, stearic acid, and stearyl alcohol were provided by Sinopharm Chemical Reagent Co., Ltd (China). The reagents of acetonitrile (chromatographic grade), anhydrous sodium hydrogen phosphate, liquid paraffin, Span 80, polysorbate 80, glycerinum, and sorbic acid were provided by Macklin (Shanghai, China). Citric acid was from Biotechnology Co. Ltd. (Tianjin, China).

### 2.2. Animals and cells

Sprague-Dawley rats (SD rats) (male, 150–180 g) were provided by Bengbu Medical College Laboratory Animal Centre. All animal experimental procedures were strictly in accordance with the guidelines for animal experiments of Bengbu Medical College and approved by the Animal Care Committee of Bengbu Medical College.

Human fibroblast synovial cells were obtained from National Collection of Authenticated Cell Cultures. The cells were cultured in Dulbecco’s modified eagle medium in an incubator at 37 °C, 5% CO_2_, and 90% relative humidity.

### 2.3. Preparation of MTX dissolving microneedle patch

The brass master mold and polydimethylsiloxane (PDMS) mold of DMNP were fabricated according to our previous study (Li et al., 2020). To prepare MTX-loaded DMNP, a two-step process was established in this study as previously described. First, 100 mg of MTX was completely dissolved in 1 mL of sodium hydroxide solution (0.04%). After adding 300 mg of HA, the solution was stirred evenly. Then, about 0.25 mL of the solution was poured into the PDMS mold and centrifuged at 3,000 rpm (Thermo Electron LED GmbH, Osterode, Germany) for 3 min. The residue solution on the surface was scraped and collected for reuse. Next, the mold was dried for 24 h in a desiccator at room temperature. Subsequently, the PVP K90 aqueous solution (25 wt%) was added into the mold and centrifuged at the same situation to fabricate the base layer. Finally, after complete desiccation, DMNP was gently detached from the molds.

### 2.4. Physical characterization of MTX-loaded DMNPs

To study the effect of excipients on MTX crystal form, X-ray diffraction (XRD) was applied to verify the structures of MTX, sodium hydroxide (material added in dissolution process), HA (needle material), the physical mixture of the three components (PM), and the microneedle powder (MNP). In addition, the chemical compositions of these components were studied by Fourier-transformed infrared spectroscopy (FTIR).

The morphology of MTX-loaded DMNPs was examined and the photos were taken by scanning electron microscopy (SEM) and an electron microscope (DM2500, Leica, Germany).

The mechanical properties of MTX-loaded DMNPs and blank DMNPs were evaluated using a texture analyzer according to previous studies (Zhao et al., 2018; Wei et al., 2022). In this experiment, the DMNP was attached to the analyzer with tape and the needles were perpendicular to the platform. Then, the probe moved to the platform. An optical microscope was used to photograph and measure the height of DMNA before and after pressure.

### 2.5. In vitro skin insertion study

To investigate insertion performance of DMNPs, in vitro skin insertion experiment was performed using rat isolated dorsal skin. The trypan blue-loaded DMNPs (0.4%) were prepared as in a previous method. After thawing and removing residual saline, the stratum corneum of skin was placed side up. Subsequently, trypan blue-loaded DMNPs were penetrated into the skin by thumb for 5 min. The insertion performance was obtained by a microscope and the penetration ratio was calculated simultaneously.

### 2.6. In vivo skin insertion and recovery tests

To investigate the in vivo insertion performance of DMNPs and skin recovery, SD rats were anesthetized with 10% chloral hydrate and shaved with an electric razor. Drug-loaded DMNPs were inserted into the skin of thigh. The rats were sacrificed at different times, and the inserte site was removed and fixed in 4% paraformaldehyde solution. Then, it was embedded in paraffin, sliced into sections, and stained by hematoxylin and eosin (H&E). The insertion depth and skin recovery were observed under the microscope.

### 2.7. In vivo dissolution

MTX-loaded DMNPs were inserted into the hairless dorsal skin and removed after different predetermined times (0, 1, 3, 5, 8, 11, 15, 20 min). The dissolution degree of microneedles was observed by a microscope. The dissolution curve was drawn by the remaining height ratio (*Y* = *I*/*I*_0_, *I*_0_ and *I* mean the initial and remaining length of the microneedles, respectively) of DMNPs at different time (*X*).

### 2.8. Preparation of MTX cream

To compare the transdermal property with DMNPs, MTX cream was prepared as follows. Briefly, stearic acid (0.6 g), stearyl alcohol (0.6 g), white valentine (0.6 g), liquid paraffin (0.9 g), and Sorbitan Oleate (0.16 g) were mixed and melted at 80 °C in a water bath to form the oil phase. The aqueous phase consisted of polysorbate 80 (0.44 g), glycerinum (1 g), and sorbic acid (0.02 g) and was heated to the same temperature. MTX (0.1 g) was dissolved in dilute alkali solution and added to the aqueous phase. Then, the aqueous phase was added to the oil phase dropwise and the mixed solution was stirred continuously until it cooled to room temperature. The final total weight should be complemented to 10 g with distilled water. Thus, the MTX cream (1%) was successfully prepared.

### 2.9. Drug content in DMNPs

The loading content of MTX in MTX-loaded DMNPs was measured by HPLC, which has gone through method validation. Drug-loaded DMNPs were separated into needles and base layer according to our previous study (Dong et al., 2020). The sample solution was obtained by dissolving needles in 1 mL of distilled water. A Shimadzu LC-16A system equipped with UV detection at the wavelength of 302 nm was used to determine drug concentration.

### 2.10. In vitro transdermal MTX delivery

In vitro transdermal MTX delivery property of DMNPs was investigated by isolated hairless dorsal skin of rats as previously described and Franz diffusion cells. MTX cream and MTX-loaded DMNPs were administrated in the isolated dorsal skin. About 0.5 mL of sample solution in the receptor chamber was collected at special time intervals (0.083, 0.25, 0.5, 1, 2, 4, 6, 8, 10, 12, and 24 h) and replaced with an equal volume of fresh PBS buffer. After filtrated by membrane, the MTX concentration of samples was determined by HPLC.

### 2.11. In vitro cytotoxicity assay

In vitro cytotoxicity of excipients and blank DMNPs were studied by MTT assay. Fibroblast synovial cells from human rheumatoid arthritis were incubated with DMEM medium containing drug-blank DMNPs, HA, and PVP at different concentrations for 48 h. MTT and DMSO were successively added into each well to form and dissolve bluish violet formazan crystals, respectively. The absorbance was determined via a microplate reader (EL800, BIO-TEK Instruments Inc., USA). The cell viability (%) was calculated according to Eq. (1).

(1) $$\text{Cell viability}(\%)=\frac{A_{\mathrm{sample}}-A_{\mathrm{blank}}}{A_{\mathrm{control}}-A_{\mathrm{blank}}}\times 100\%$$

### 2.12. In vivo pharmacodynamic study

#### 2.12.1. AIA model and arthritis assessment

AIA model was established to evaluate the therapeutic potency of MTX-DMNs in vivo. Bacillus Calmette-Guerin was thoroughly ground with the sterile liquid paraffin to produce complete Freund adjuvant (CFA, 10 mg/mL). The rats were injected subcutaneously at the right hind paw with 80 μL of CFA to build AIA model on day 0. On day 21, the successful modeling of rats was randomly divided into four groups: model group, MTX cream group, MTX oral administration group, and MTX-loaded DMNPs group. Normal rats without induced by CFA were put in normal group (*n* = 6). The drug groups were administrated MTX (0.2 mg) at different formulations every there days for seven times. DMNPs were inserted into the prepared hairless skin of thigh by thumb and immobilized for 30 min by medical tape to ensure the complete dissolution of needles.

During the treatment, the global symptoms of rats were observed and the swelling volume of hind paw of rats in each group was determined by a paw volume meter before immunization and every three days after treatment with drug. The changes in paw volume were calculated according to the following formula: posterior paw swelling volume (mL) = *V*_t_ – *V*_0_, where *V*_0_ was the volume before CFA immunization and *V*_t_ was the volume on day t after CFA immunization.

#### 2.12.2. Radiological examination

The severity of swelling of soft tissue and narrowing of joint space was measured using radiological examination. After the last dose, the rats were anesthetized and subsequently performed the examination of ankle and hind limb by X-ray imaging.

#### 2.12.3. Histological examinations and immunohistochemical of synovium

After blood collection, the rats were sacrificed on day 45. The synovium of ankle join was immediately removed and fixed in 4% paraformaldehyde for further study. After one week, the fixed tissue was embedded and sliced to sections, which were stained with H&E and observed by optical microscopy for histopathological analysis.

Meanwhile, the sliced samples were labeled with protein according to the immunohistochemical protocol. After DAB staining, the brown area was observed as positive expression under the microscope to analyze the immunohistochemical result.

#### 2.12.4. Cytokine measurement by ELISA

To determine the serum cytokine level, blood samples (3.5 mL) were collected from the oculi vein after last drug administration. After standing at 4 °C for 1 h, the samples were centrifuged at 5000 rpm for 15 min to obtain the supernatant serum and stored for biochemical assay. The serum level of interleukin-1beta (IL-1β) and TNF-α were measured using enzyme-linked immunosorbent assay (ELISA, RayBiotech, Norcross, GA) in accordance with the manufacturer’s protocol.

### 2.13. Statistical analysis

The data were performed by GraphPad Prism software (Version8.0.2) with one-way ANOVA and expressed as the mean ± standard deviation. *p* < .05 was considered statistically significant, and *p* < .01 represented extremely significant.

## 3 Results and discussion

### 3.1. Preparation and morphology of DMNPs

**Figure 2.**
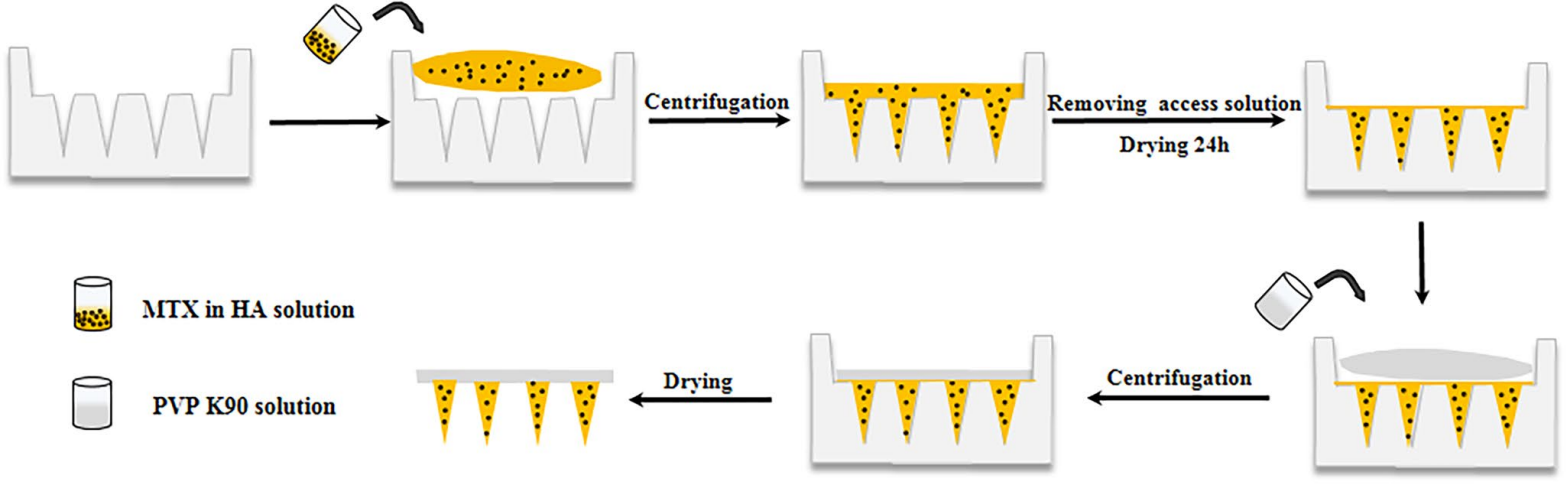
Fabrication of MTX-loaded DMNPs by two-step process.

MTX-loaded DMNPs were prepared using micromolding with a two-step process as shown in Figure 2. When prescription screening, excipients including chondroitin sulfate (CS), dextran (DEX), and hyaluronic acid (HA) were selected to prepare MTX-DMNPs as excipients of needles. The results showed that the needles of DMNPs prepared by CS or HA were complete and rectangular in order, while DEX-prepared ones were defective or easy to bend, indicating that DEX was not suitable to fabricate MTX-loaded DMNPs (Supplementary Figure S1(A–C)).

**Figure 3.**
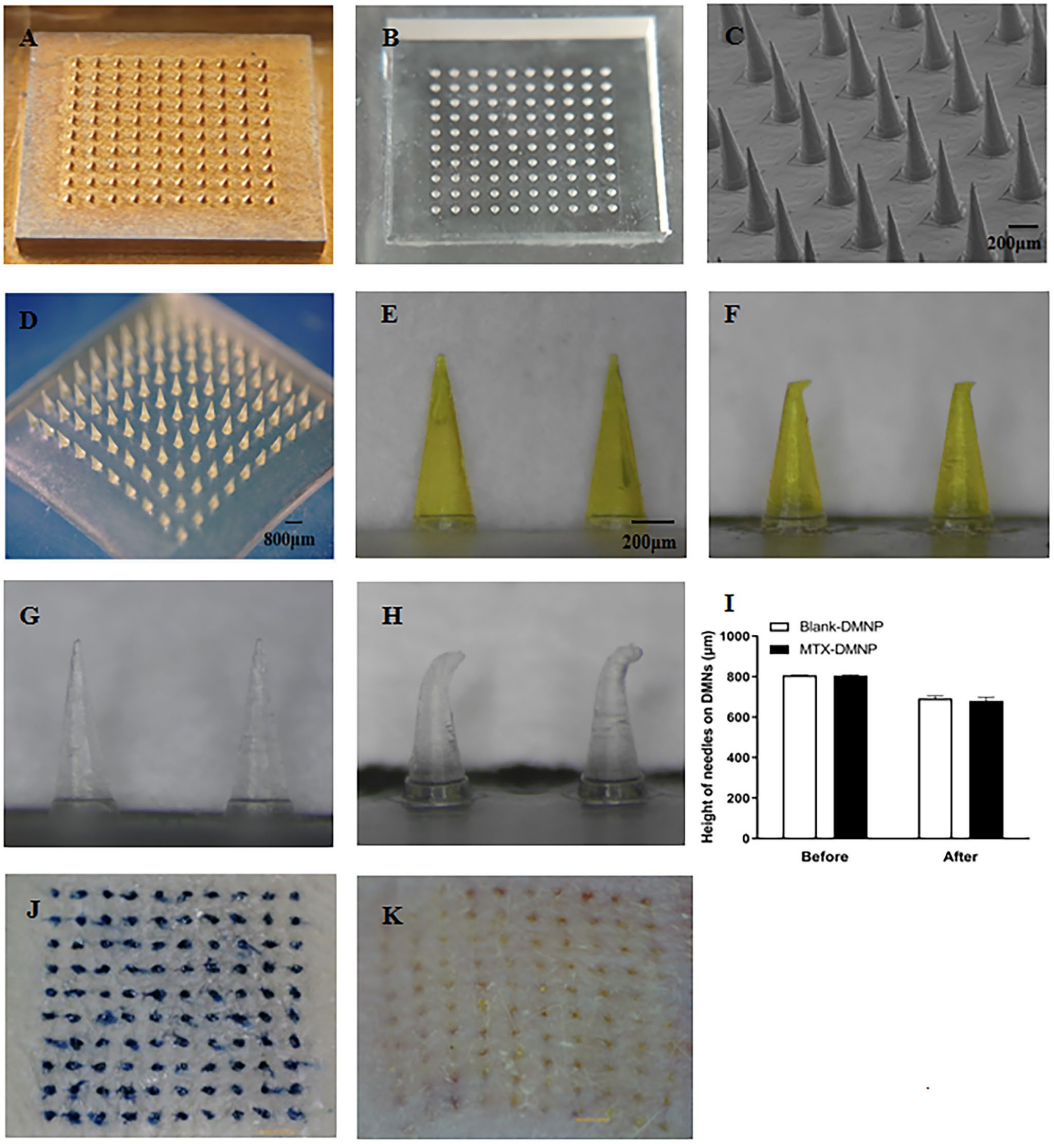
Photographs of microneedle master mold (A) and PDMS female mold (B), scanning electron microscopy (SEM) image (C), and the microscope images (D and E) of MTX-loaded DMNPs, the microscope image of MTX-loaded DMNP after compression (F), the microscope images of blank DMNP before (G) and after (H) compression. The height of DMNPs before and after compression (I). Image of isolated rat skin penetrated by TB-loaded DMNP (J), Data are presented as the mean ± SD (*n* = 5). In vivo insertion study in rats using MTX-loaded DMNP (K).

As shown in Figure 3(C), the prepared MTX-loaded DMNPs consisted of 100 microneedles arranged in 10 × 10 arrays. The optical microscope and scanning electron microscope (SEM) were applied to observe more detailed morphology (Figure 3(D,E)). The microneedles had uniform conical shape and size with 800 μm in height, 300 μm diameter at bottom, and 900 μm distance from pinpoint to pinpoint. Under microscopes, it was clear that the morphology of DMNPs was conical array with sharp tip and smooth surface of the needle.

### 3.2. Mechanical properties of DMNPs

The morphology of MTX-loaded and blank DMNPs before and after pressure was shown in Figure 3(E–H). It showed that the microneedles were bent rather than broken after the application of 100 N for 5 s, which meant that the DMNPs had a certain degree of compression resistance for skin insertion. The change of height after pressure was also observed (Figure 3(I)). The height of blank DMNPs and MTX-loaded DMNPs decreased by 14.2% and 15.6% respectively without no statistical significance. Therefore, the addition of MTX did not affect the mechanical properties of DMNP.

In order to study drug and excipients compatibility, FTIR and XRD were used to analyze the structure and morphology of MTX and auxiliary materials, respectively. Considering that a two-step process was used to prepare MTX-loaded DMNPs, it was mainly studied the interaction between MTX and needle materials.

The FTIR spectrum of MTX (Supplementary Figure S1(D)) showed that the characteristic band of MTX at 3300–3400 cm^−1^ is the stretching vibration of N-H. The characteristic band at 1641 cm^−1^ corresponded to -CONH. The characteristic band at 1601 cm^−1^ belonged to the C = C stretching vibration of the benzene ring, while the band at 1496 cm^−1^ was due to the symmetric stretching mode of COO-. The peaks centered at 1208 cm^−1^ represented C-N stretching. Remarkably, the characteristic spectrum of MTX was also existed in PM and MNP. Therefore, the chemical structure of MTX did not alter during the preparation of microneedles.

As shown in Supplementary Figure S2(E), the effect of excipients on the crystal state of MTX was further studied by XRD analysis. The characteristic peaks of MTX were still present in PM and MNP, indicating that the addition of HA and sodium hydroxide did not affect the crystal form of MTX. Meanwhile, the characteristic peak of sodium hydroxide in the mixture was not obvious, which may be due to the tiny proportion.

### 3.3. Drug content

It had been validated that HPLC-UV method was reliable for the determination of MTX content in DMNPs, with good linearity in the concentration range of 5–220 μg/mL (*R*^2^ = 0.9998). The retention time of MTX was 6.2 min without interference from excipients (Supplementary Figure S2). The intra-day and inter-day average precisions were 0.7 and 0.97 respectively (Supplementary Table S1). The results demonstrated a good recovery in the range of 95.19%–−101.07% with RSD < 2% (Supplementary Table S2).

The excipients of DEX, HA and CS were chosen to prepared MTX-loaded DMNPs and the drug loading was detected by HPLC. The results showed that the drug contents of the needle part in three prescriptions of MTX-loaded DMNPs were 82.33 ± 3.36, 205.08 ± 3.77, and 167.67 ± 2.07 μg, respectively (*n* = 3, Supplementary Figure S1(F)). Considering the needle shape and needle loading capacity of MTX-DMNPs, HA was selected as the needle material of DMNPs for subsequent study.

### 3.4. Skin insertion study

To examine whether the microneedles could successfully penetrate the skin, in vitro and in vivo insertion experiments were performed. In in vitro experiments, the isolated dorsal skin of SD rats was penetrated using TB-loaded DMNPs for 5 min and removed afterward. Trypan blue was adopted because it stains internal tissues of skin through micropores and could not penetrate the *SC* itself (Cheng et al., 2020). Therefore, the conspicuous blue dots in isolated skin observed by a handheld microscope could indicate the puncture capability (Figure 3(J)). The effect of different concentrations of HA on puncture performance was also studied (Supplementary Figure S3). The results showed that the insertion ratios of 100 and 200 mg/mL HA were about 72% and 85% respectively, while the ratios of 300 mg/mL HA were more than 95%, which was therefore adopted to fabricate DMNPs for further studies.

**Figure 4.**
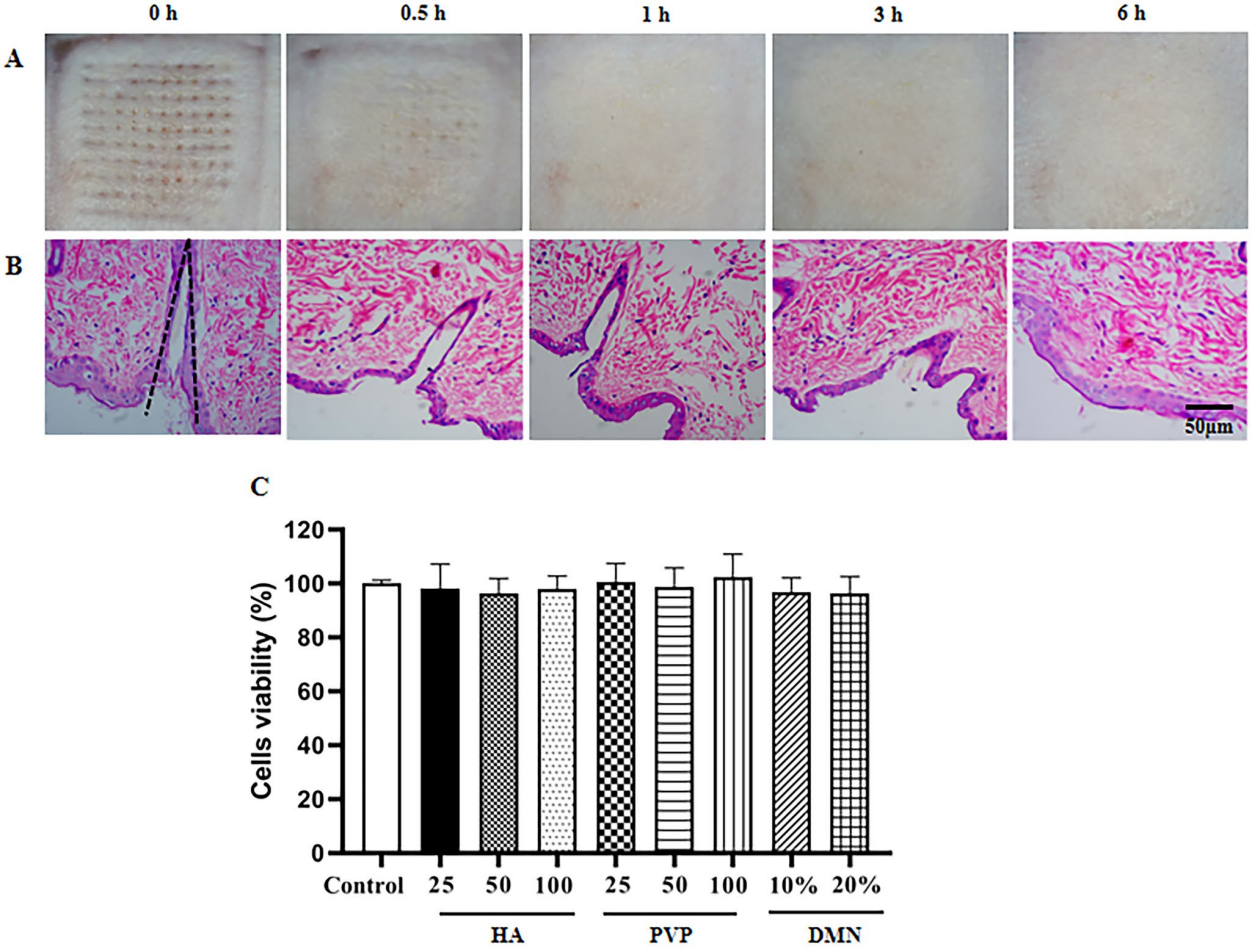
In vivo skin insertion and safety evaluation of MTX-loaded DMNPs. Skin recovery images (A) and H&E staining of inserted skin sections (B) at 0, 0.5 h, 1 h, 3 h, and 6 h after DMNPs treatment. (C) MTT assay of blank DMNPs and materials which constituted DMNPs solution at different concentrations for 48 h (Mean ± SD, *n* = 5).

The puncture performance in vivo and skin recovery were also investigated in rats using MTX-loaded DMNPs. As shown in Figure 3(K), the results exhibited that the array of microneedles formed on the skin was clearly visible, which was consistent with in vitro results. To further illustrate the skin puncture capability in vivo, this piece of skin was quickly stripped and stained with H&E to observe the histological morphology under microscope. It revealed that DMNs punctured the skin with about 200 μm depth and formed regular cone-like pinhole morphology in the skin (Figure 4(B)). According to previous study (Ito et al., 2013), the results proved DMNs we prepared could effectively penetrate the SC without touching nerves or blood vessels in the dermis.

In the experiment of skin recovery (Figure 4(A,B)), the administration skin areas of rats were photographed at different time after applied with MTX-loaded DMNPs. Then, the administrated skin was sectioned and stained with H&E as before. The results indicated that the skin after administration recovered gradually and the aperture formed by DMNPs decreased with time going. Interestingly, it seemed that the skin had recovered at 1 h, while the pathologic results revealed that the inner tissue recovered completely after 6 h. However, some studies have reported that the rapid visual recovery of microneedles may not have healed at the pathological level.

### 3.5. In vitro cytotoxicity assay

To evaluate the bio-safety of excipients, fibroblast synovial cells were incubated with blank DMNPs or mixture of excipients and cell viability was detected via MTT assay. The results showed that more than 80% of cells were viable at experimental concentrations (Figure 4(C)), which indicated that both the materials required for the preparation of microneedles and blank DMNPs showed almost low toxicity and favorable biocompatibility.

### 3.6. In vivo dissolution

**Figure 5.**
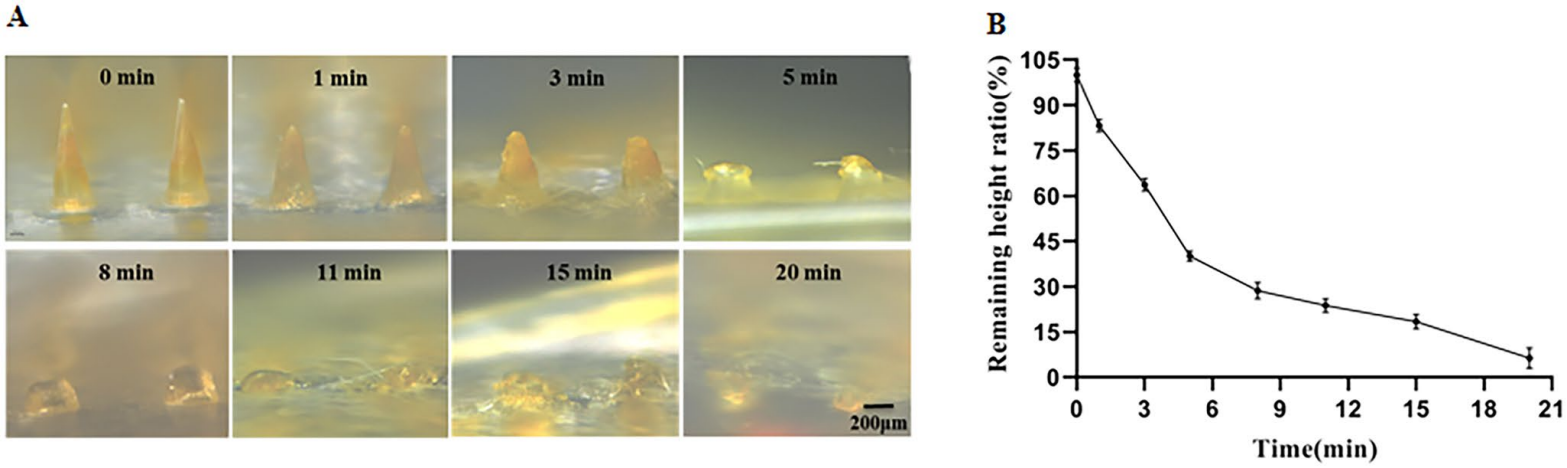
Dissolution of MTX-loaded DMNPs in vivo. (A) Microscope images of DMNPs after insertion for different time in vivo. (B) Dissolution curve of MTX-loaded DMNPs in vivo (*n* = 5).

To further determine the dissolution time of MTX-loaded DMNPs in skin, the remaining height of the microneedles were observed by a microscope after being pressed on the legs of rats for different time (Figure 5(A)), and the dissolution curves were shown in Figure 5(B). The results showed that DMNPs dissolved rapidly in the first 5 min and subsequently the dissolution rate slows down. Finally, the needles of MTX-loaded DMNPs were almost completely dissolved within 20 min. The drug could be released from the microneedles into the skin to work.

### 3.7. In vitro transdermal MTX delivery

**Figure 6.**
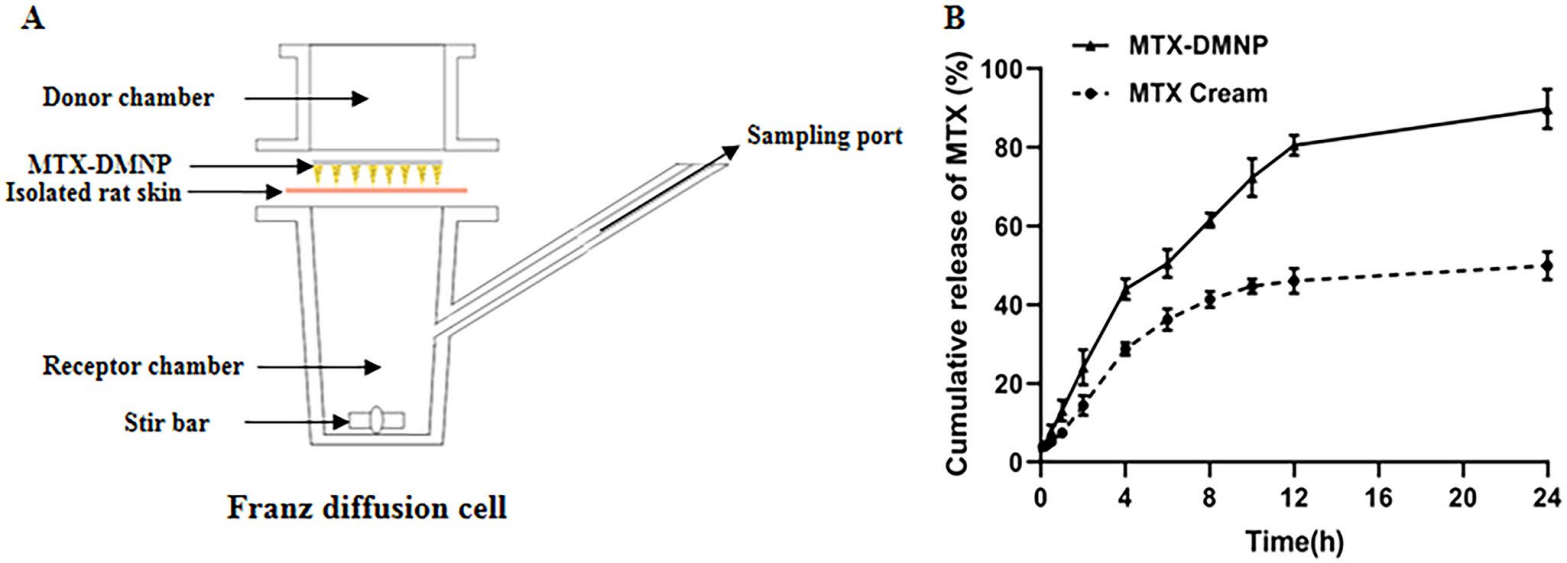
In vitro transdermal delivery of MTX. (A) Schematic illustration of Franz diffusion cell. (B) In vitro transdermal delivery profile of MTX from DMNs and cream in isolated rats skin. Data are presented as the mean ± SD (*n* = 6).

To study and compare the transdermal delivery performance of MTX cream and MTX-loaded DMNPs, the isolated hairless dorsal skin of rats and Franz diffusion cells (Figure 6(A)) were applied. The cumulative transdermal release ratio (%) –time (h) curve of MTX was plotted. As shown in Figure 6(B), the cumulative release amount of MTX in DMNs group was dramatically higher than that of MTX cream. After 24 h, the release curves of the two groups tended to equilibrium, and about 89.80% and 49.92% of MTX were released into the receptor chamber from DMNP group and cream group, respectively. The results indicated that microneedles could significantly increase drug transdermal permeation compared with the cream group.

### 3.8. In vivo pharmacodynamic study

#### 3.8.1. Arthritis evaluation and paw swelling degree

**Figure 7.**
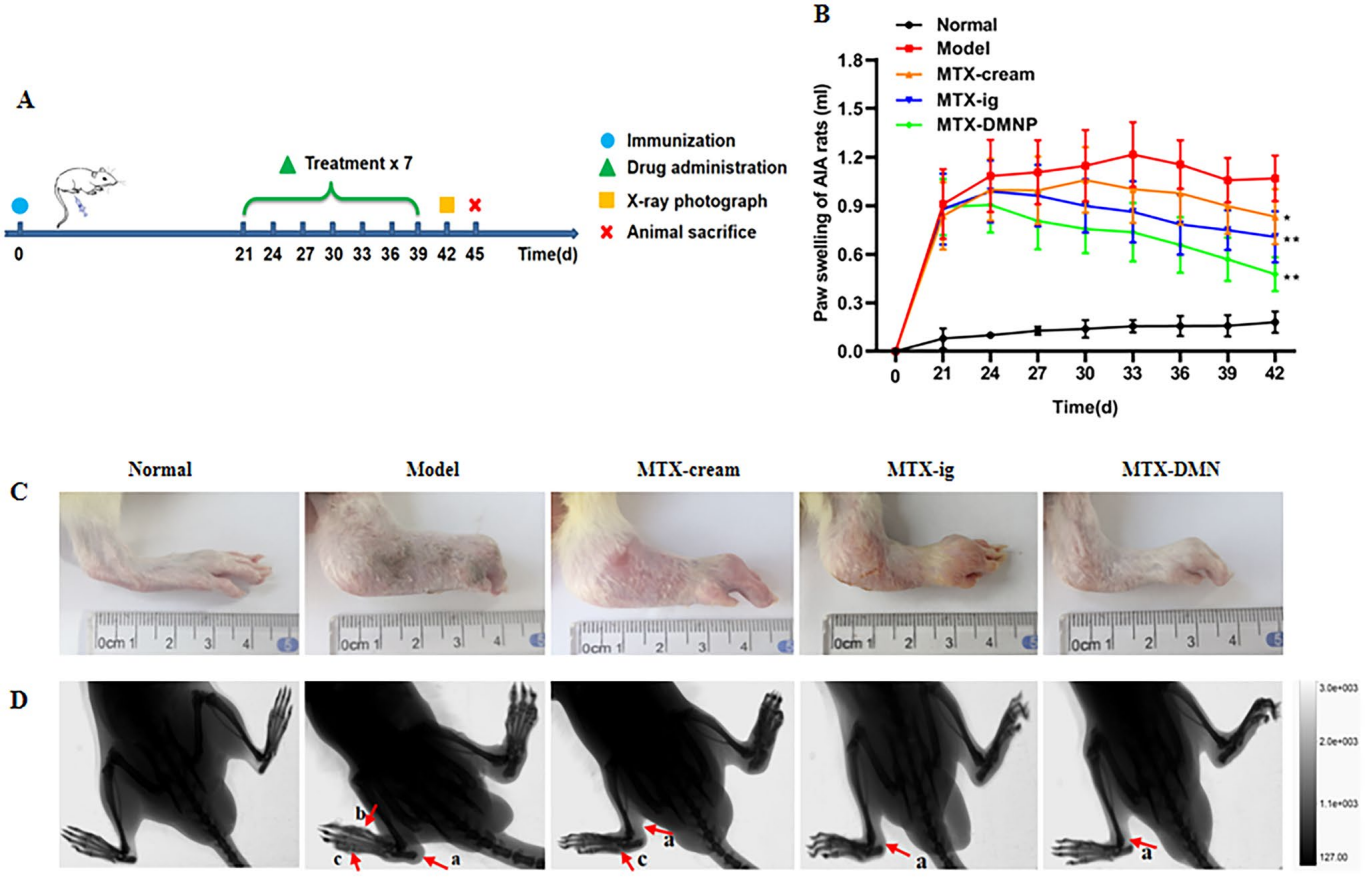
Therapeutic potency of MTX-loaded DMNs in rats. (A) Experimental design for therapeutic potency study. (B) Changes of left paw volume of different groups during treatment (mean ± SD, *n* = 6). *p < .05, **p < .01 compared with Model group. (C) The left hind paw images of rats from different groups on day 42. (D) X-ray radiological images of rat paws from different groups on day 42 (a: soft tissue swelling, b: bony density reduction, c: bony defect).

AIA model is always applied in pharmacodynamic study as it has similar immunological and pathological features with human (Grötsch et al., 2019). Early local inflammation appeared in SD rats after being immunized with CFA 1–3 days, resulting in slight redness and swelling of the modeling lateral claw, which lasted for 2–4 days and gradually subsided. After about 10 days of immunization, the modeling lateral claw of AIA rats swelled again. On day 15, AIA rats began to appear secondary inflammatory response. With the progress of inflammation, redness, and swelling of the ankle joints aggravated to the metacarpal or toe joint, and redness or nodules appeared in the nose, ears, and tail of AIA rats. The occurrence of secondary inflammation marked the successful induction of AIA model by CFA. After 19 days of immunization, the rats entered the peak of systemic multiple joint inflammation, which presented dull hair, dysfunctional joint, and even ankle ulceration in serious cases. According to the above evaluation criteria, the successful modeling of rats was randomly divided into different groups to study anti-arthritis effect of MTX-loaded DMNPs. The experimental schedule of the in vivo pharmacodynamic study was shown in Figure 7(A).

Paw thickness could reflect joint swelling and synovial inflammation to a certain extent. Therefore, the swelling volume of left hind paw of rats was determined by a paw volume meter every three days and the results are presented in Figure 7(B). After successful modeling, the swelling degree of left hind paw in model group without administration was markedly higher than normal group (*p* < .01). Compared with model group, the paw thickness of rats treated with drug formulations was markedly decreased after the last administration (cream group *p* < 0.05, oral and DMNP *p* < 0.01), indicating that long-term MTX treatment reduced the paw swelling degree and relieved inflammation. Simultaneously, the paw swelling degree among different MTX preparations was also analyzed. The swelling volume of MTX DMNP group was significantly reduced compared to that of cream group (*p* < 0.01) or oral group (*p* < 0.05) at the same dosage of MTX. The paw images further confirmed that the paw swelling of RA rats treated with MTX formulations were significantly relived on day 42 (Figure 7(C)). The cream group and oral group still showed obvious redness and swelling of hind paw. However, DMNP group, which had the closest condition to the control group, presented better therapeutic effect as compared to cream or oral group. In brief, the results showed that the AIA rats treated with 200 μg MTX-loaded DMNP could effectively inhibit the paw swelling of rats, which was better than other groups.

#### 3.8.2. Radiological examination

X-ray was a convenient method to observe the anatomical structures of joints as it could provide detailed information in a noninvasive way in vivo. After 21 days of different treatments, the radiological changes of hind limbs of rats were examined by X-ray. In normal group, the rats had complete and healthy joint structure without swelling (Figure 7(D)). In contrast, the model group presented severe soft tissue swelling, reduced local bone density and bone destruction. However, the pathological changes of hind paws of rats treated with MTX preparations were significantly improved. The cream group showed obvious soft tissue swelling and bone defect, and the oral group and DMNP group mainly showed swelling. It was also observed that the oral group had more serious swelling than DMNP, which was consistent with the result of paw volume. The results indicated that MTX drug therapy could effectively alleviate the above lesions and relieve inflammation in AIA rats. Among different preparations, MTX DMNPs showed better therapeutic effect than other groups in relieving the pathological changes of hind paws of AIA rats.

#### 3.8.3. Histological examination of synovium

**Figure 8.**
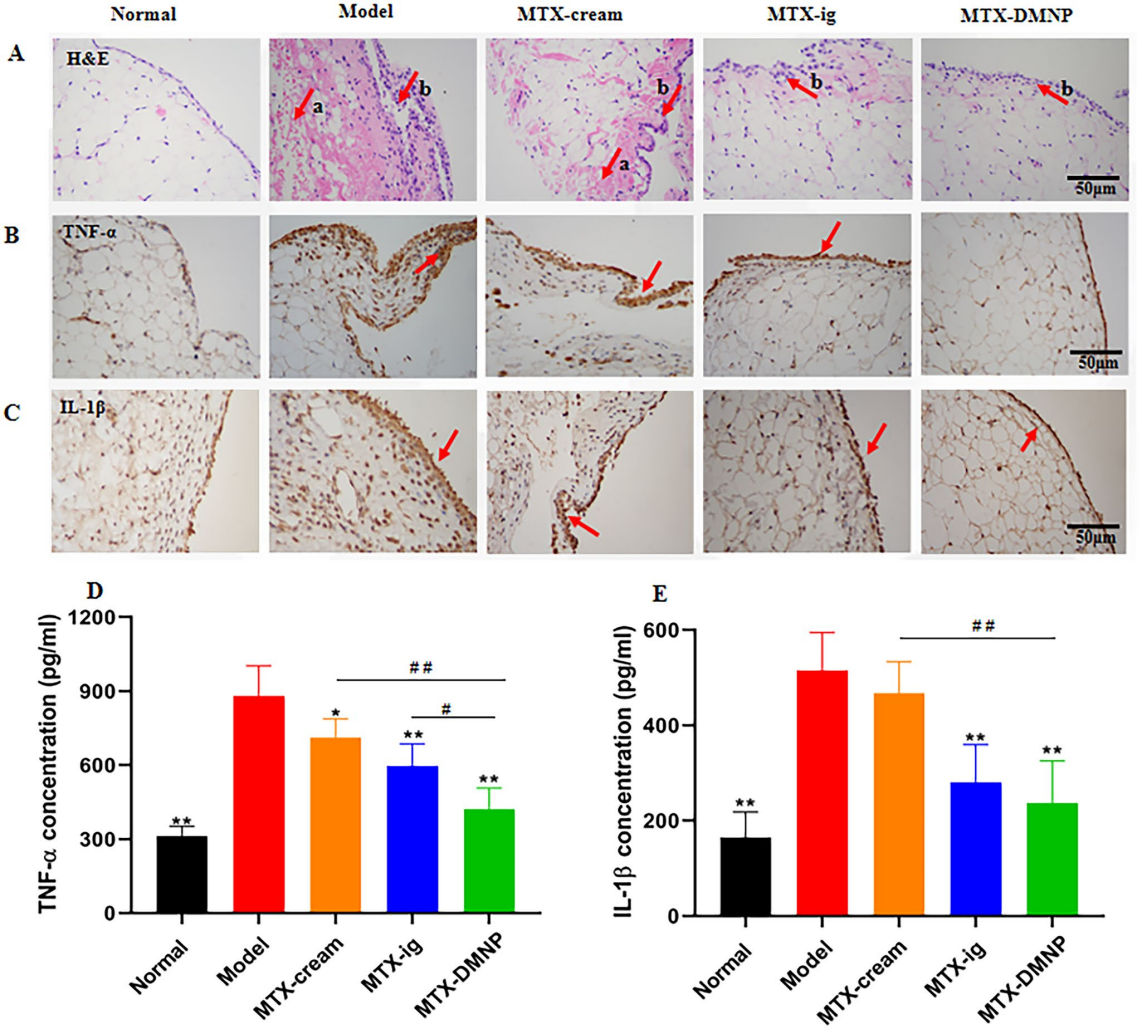
H&E staining (A) and immunohistochemistry of TNF-α (B) and IL-1β (C) in rat synovium from different groups (a: pannus formation, b: synovial proliferation). TNF-α (D) and IL-1β (E) levels of rat serum in different groups (mean ± SD, *n* = 5), *p < .05, **p < .01 compared with Model group, #*p* < .05, ##*p* < .01 compared with MTX-DMNP group.

After the last dose of administration, the synovial of rat ankle joints in all groups was sectioned, stained by H&E and observed under microscope. Compared with the normal group, the rats in model group showed synovial cells hyperplasia and disorganized arrangement, pannus formation, and obvious inflammatory cell infiltration (Figure 8(A)). MTX drug therapy can reduce inflammatory lesions and relieve inflammation, indicating that MTX effectively alleviated synovium destruction, which was consistent with previous studies. DMNP group depicted less synovial cells hyperplasia and pannus formation than cream and oral group. The HE staining images of DMNP group were similar to normal group. The results showed the synovium in the rats treated with MTX-loaded DMNPs was alleviated considerably and better than other groups.

#### 3.8.4. Immunohistochemical of synovium

The expression of TNF-α and IL-1β in synovium was observed via immunohistochemical assay. In this experiment, after colored by DAB, the brown area was observed under the microscope, that is, the size of the brown area represented the level of labeled protein expression. The expression of TNF-α and IL-1β in synovium in model group was markedly higher than normal rats (Figure 8(B,C)). However, the expression of above cytokines in all drug groups presented marked decrease as compared to the model group. It indicated that TNF-α and IL-1β expression in synovial tissue of AIA rats increased significantly after inflammation, and MTX drug therapy could reduce pro-inflammatory cytokines expression and thus relieve inflammation. At the same dosage of MTX, DMNP group showed significant decrease compared with cream or oral group, which meant that DMNP presented the best therapeutic effect.

#### 3.8.5. Cytokine measurement by ELISA

The level of cytokines was one of the important factors leading to synovial inflammation and osteodestruction of articular cartilage. The pro-inflammatory cytokines secreted by immune cells, such as TNF-α and IL-1β, recruited a large number of neutrophils, influenced the differentiation of T cells and stimulated the abnormal activation and proliferation of the fibroblast-like synovial cells (FLS) and thus enhanced the inflammatory response, resulting in the damage of synovial tissue. Therefore, the serum level of pro-inflammatory cytokines determined by ELISA kit was often used to evaluate the efficacy of RA treatment. TNF-α and IL-1β are frequently used to evaluate RA treatment. IL-1β is a vital cytokine that promotes inflammation, regulates chondrocyte apoptosis and accelerates joint destruction via increasing osteoclast absorption (Rzepecka et al., 2015). Likewise, TNF-α plays an important role in immunomodulation, systemic, and local inflammation (Solomon et al., 2013).

As shown in Figure 8(D,E), the serum levels of TNF-α and IL-1β, were significantly increased in model group compared with normal rats (*p* < .01), suggesting that cytokines content in the AIA model rats was closely related to the progression of RA. The serum levels of TNF-α and IL-1β in AIA rats treated with different MTX formulations were decreased, especially in oral and DMNP group. Compared with cream group, the concentrations of TNF-α and IL-1β in MTX DMNP group were significantly reduced (*p* < .01), which may be attributed to the penetration promotion of MTX by DMNP. Likewise, DMNP group showed significant decrease level of TNF-α (*p* < .05) compared to oral group. The level of IL-1β in DMNP group was lower than that of oral group, but there was no significance difference, which may be due to the low concentration and considerable individual differences. In conclusion, MTX-loaded DMNPs could alleviate inflammation of RA effectively by decreasing the serum concentrations of pro-inflammatory cytokines.

## 4. Conclusion

As far as we know, there was few reports about the effect of MTX-loaded dissolving microneedles on RA. The delivery of MTX via microneedles was mainly studied for psoriasis treatment (Tekko et al., 2020b) or only for preparation evaluation (Tekko et al., 2020a). In this study, we have innovatively developed a novel dissolving microneedles to deliver MTX for RA therapy. It has excellent morphology with neat array and complete needles, optimal mechanical strength enough to pierce the skin, stable drug loading, and fast intradermal dissolution. Furthermore, it has been demonstrated that the DMNPs could effectively penetrate the stratum corneum and significantly increase MTX transdermal permeation. It was also found that MTX-loaded DMNs significantly alleviated paw swelling, inhibited inflammation via reducing the level of pro-inflammatory cytokines such as TNF-α and IL-1β, and relieved synovium destruction with less cartilage erosion. Compared with cream or conventional oral administration, transdermal delivery of MTX by DMNs presented better therapeutic performance at same dosage. In conclusion, the MTX-loaded HA dissolving microneedle patch we prepared in this study has advantages of safety, convenience and high efficacy over conventional administrations, making it a promising therapy for the treatment of RA.

## Supplementary Material

Supplemental Material
