# Supplementary material for: Dissolving microneedle patch-assisted transdermal delivery of methotrexate improve the therapeutic efficacy of rheumatoid arthritis
Source: Drug Deliv. 2022 Dec 19;30(1):121–32. doi: 10.1080/10717544.2022.2157518 (PMC9769132; doi:10.1080/10717544.2022.2157518)
Supplement: Supplemental Material [file IDRD_A_2157518_SM0983.docx]

**Supporting information**


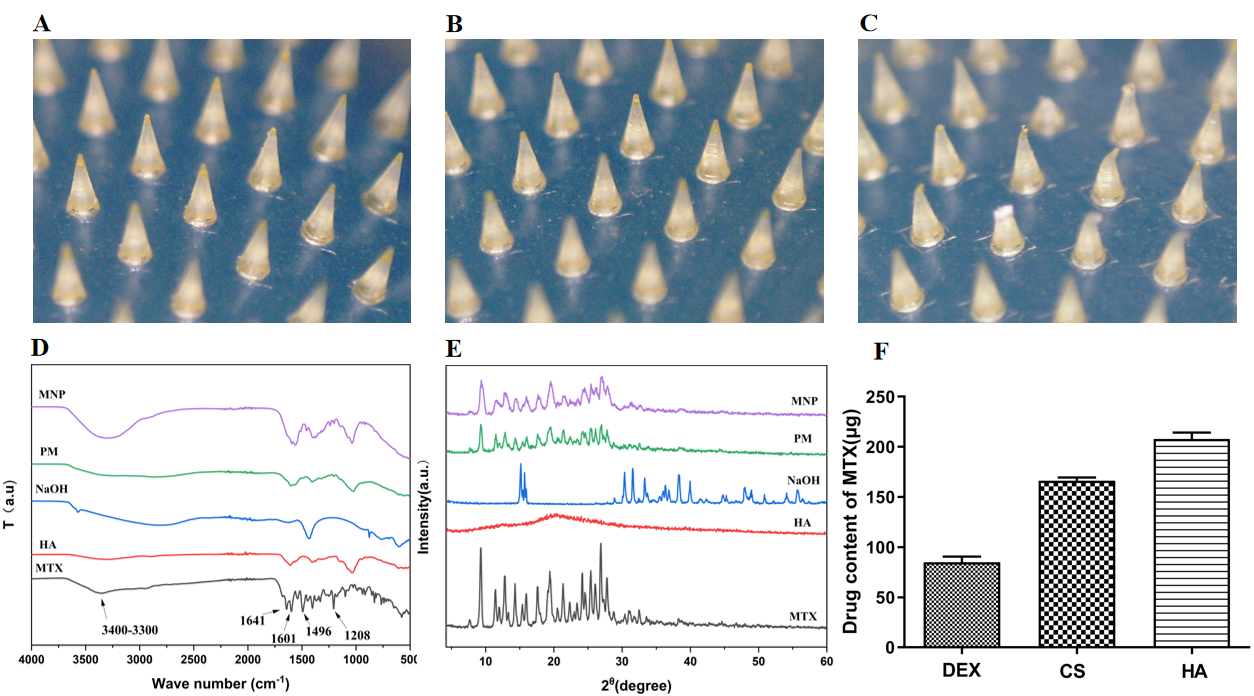


Figure S1 The microscope images of MTX-loaded DMNs with different excipients :(A)HA, (B)CS, (C)DEX; (D)FTIR of MTX, HA, NaOH, PM and MNP; (E)XRD of MTX, HA, NaOH, PM and MNP; (F)MTX content of needles in DMNs with different formulations.

Table S1 Intra- and inter-day precision of determination of MTX in DMNPs(n = 3)

| Conc.  (μg /mL) | Mean(SD) | | %RSD | | %Accuracy | | |  |
| --- | --- | --- | --- | --- | --- | --- | --- | --- |
|  | Intra-day | Inter-day | Intra-day | Inter-day | | Intra-day | Inter-day | |
| 10 | 9.35（0.06） | 9.32（0.09） | 0.62 | 0.97 | | 94.43 | 94.19 | |
| 100 | 101.95（0.98） | 101.35（0.32） | 0.96 | 0.32 | | 102.97 | 102.37 | |
| 200 | 191.03（1.00） | 188.74（3.09） | 0.52 | 1.63 | | 96.48 | 95.33 | |

Table S2 Results of recovery test of MTX(n = 3)

| Added Conc.  (μg /mL) | Detected Conc.  （μg/mL） | Recovery  （%） | Average Recovery  （%） | RSD  （%） |
| --- | --- | --- | --- | --- |
| 19.8 | 19.12 | 96.56 | 97.84 | 1.97 |
|  | 19.16 | 96.78 |  |  |
|  | 19.84 | 100.18 |  |  |
| 39.6 | 38.52 | 97.27 | 96.59 | 1.22 |
|  | 37.70 | 95.19 |  |  |
|  | 38.54 | 97.31 |  |  |
| 79.2 | 78.27 | 98.82 | 100.32 | 1.27 |
|  | 80.05 | 101.07 |  |  |
|  | 80.04 | 101.06 |  |  |

A

B

D

C

E

F

G

Figure S2 HPLC chromatograms of (A). blank solution; (B). CS; (C). DEX; (D). HA; (E). PVP K90; (F). MTX; (G). MTX-loaded DMNP


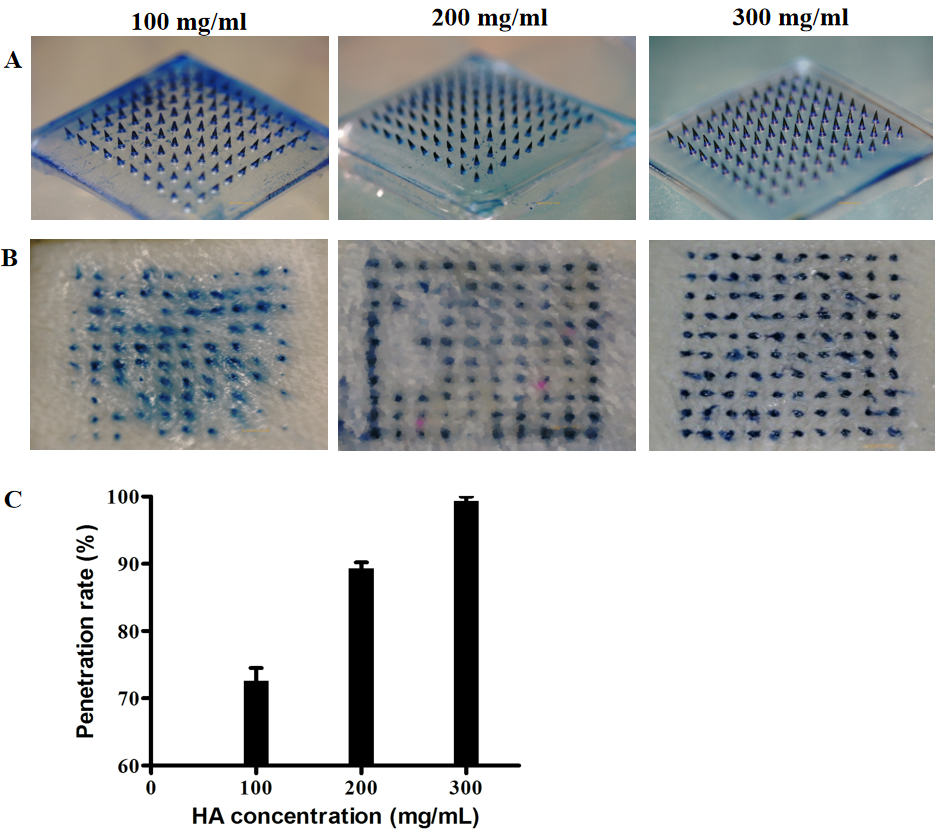


Figure S3 (A)TB-loaded DMNs , (B) skin insertion images and (C) penetration rate with different HA content in isolated dorsal skin of SD rats.
